# Supplementary figures and images for: Zinc finger protein Zfp335 controls early T-cell development and survival through β-selection-dependent and -independent mechanisms
Source: eLife. 2022 Feb 3;11:e75508. doi: 10.7554/eLife.75508 (PMC8871394; doi:10.7554/eLife.75508)

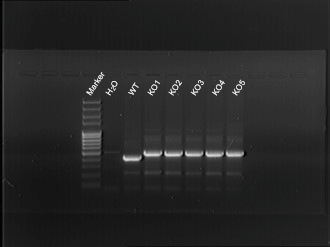

Supplement: Figure 1—figure supplement 3—source data 1. — Lines 1–8: 1: marker; 2: H2O; 3: WT; 4: KO1; 5: KO2; 6: KO3; 7: KO4; 8: KO5. [file elife-75508-fig1-figsupp3-data1.zip › Figure 1-figure supplement 3-source data 1/Figure 1-figure supplement 3-source data 1_raw gel picture with labels.tif]

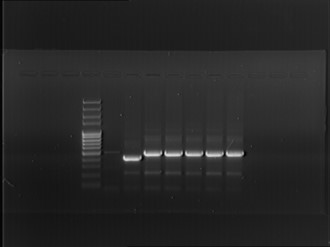

Supplement: Figure 1—figure supplement 3—source data 1. — Lines 1–8: 1: marker; 2: H2O; 3: WT; 4: KO1; 5: KO2; 6: KO3; 7: KO4; 8: KO5. [file elife-75508-fig1-figsupp3-data1.zip › Figure 1-figure supplement 3-source data 1/Figure 1-figure supplement 3-source data 1_raw gel picture.tif]

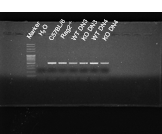

Supplement: Figure 5—figure supplement 3—source data 1. — Lines 1–8: 1: marker; 2: H2O; 3: WT; 4: Rag2−/−; 5: WT DN3; 6: KO DN3; 7: WT DN4; 8: KO DN4. [file elife-75508-fig5-figsupp3-data1.zip › Figure 5-figure supplement 3-source data 1/Figure 5-figure supplement 3-source data 1_raw gel picture with labels.tif]

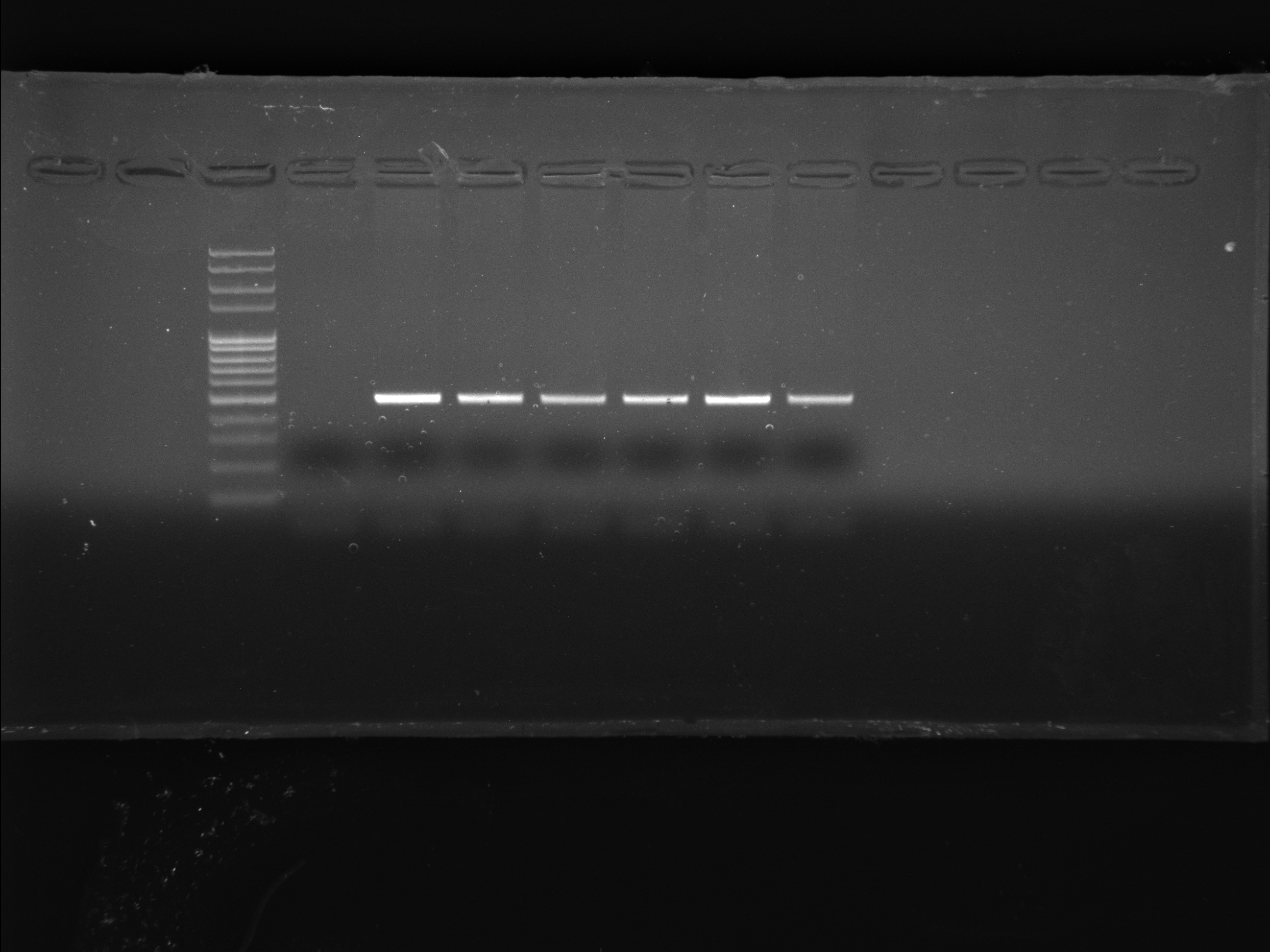

Supplement: Figure 5—figure supplement 3—source data 1. — Lines 1–8: 1: marker; 2: H2O; 3: WT; 4: Rag2−/−; 5: WT DN3; 6: KO DN3; 7: WT DN4; 8: KO DN4. [file elife-75508-fig5-figsupp3-data1.zip › Figure 5-figure supplement 3-source data 1/Figure 5-figure supplement 3-source data 1_raw gel picture.tif]

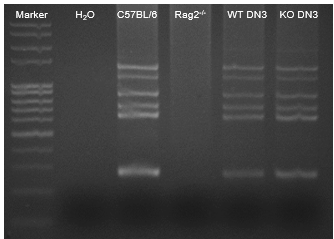

Supplement: Figure 5—figure supplement 3—source data 2. — Lines 1–6: 1: marker; 2: H2O; 3: WT; 4: Rag2−/−; 5: WT DN3; 6: KO DN3. [file elife-75508-fig5-figsupp3-data2.zip › Figure 5-figure supplement 3-source data 2/Figure 5-figure supplement 3-source data 2_raw gel picture with labels.tif]

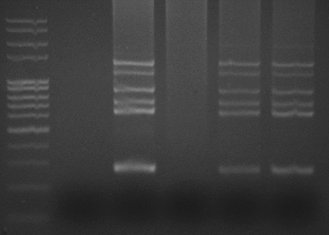

Supplement: Figure 5—figure supplement 3—source data 2. — Lines 1–6: 1: marker; 2: H2O; 3: WT; 4: Rag2−/−; 5: WT DN3; 6: KO DN3. [file elife-75508-fig5-figsupp3-data2.zip › Figure 5-figure supplement 3-source data 2/Figure 5-figure supplement 3-source data 2_raw gel picture.tif]

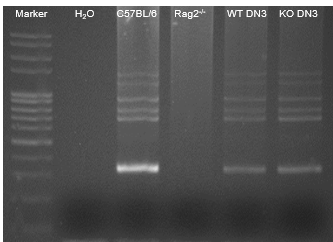

Supplement: Figure 5—figure supplement 3—source data 3. — Lines 1–6: 1: marker; 2: H2O; 3: WT; 4: Rag2−/−; 5: WT DN3; 6: KO DN3. [file elife-75508-fig5-figsupp3-data3.zip › Figure 5-figure supplement 3-source data 3/Figure 5-figure supplement 3-source data 3_raw gel picture with labels.tif]

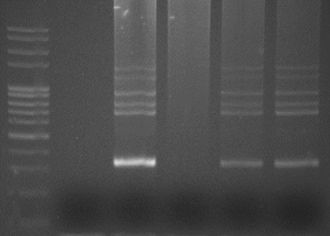

Supplement: Figure 5—figure supplement 3—source data 3. — Lines 1–6: 1: marker; 2: H2O; 3: WT; 4: Rag2−/−; 5: WT DN3; 6: KO DN3. [file elife-75508-fig5-figsupp3-data3.zip › Figure 5-figure supplement 3-source data 3/Figure 5-figure supplement 3-source data 3_raw gel picture.tif]
